# Liposomal Bupivacaine (Bupigel) Demonstrates Minimal Local Nerve Toxicity in a Rabbit Functional Model

**DOI:** 10.3390/pharmaceutics13020185

**Published:** 2021-02-01

**Authors:** Yaelle Bavli, Malcolm Rabie, Yakov Fellig, Yoram Nevo, Yechezkel Barenholz

**Affiliations:** 1 Laboratory of Membrane and Liposome Research, Department of Biochemistry, IMRIC, The Hebrew University-Hadassah Medical School, Jerusalem 9112102, Israel; yaellef@ekmd.huji.ac.il; 2 Institute of Neurology, Schneider Children’s Medical Center of Israel, Tel-Aviv University, Petach Tikva 4920235, Israel; malcolmr1209@gmail.com (M.R.); yoramne@clalit.org.il (Y.N.); 3 Pediatric Neuromuscular Laboratory, Felsenstein Medical Research Center, Tel-Aviv University, Petach Tikva 4920235, Israel; 4 Department of Pathology, The Hebrew University-Hadassah Medical School, Jerusalem 9112102, Israel; fellig@hadassah.org.il

**Keywords:** nerve conduction study, New Zealand White (NZW) rabbits, neurotoxicity, liposomes, long acting local anesthetic

## Abstract

We previously reported the development of a novel formulation of an ultra-long-acting local anesthetic based on bupivacaine encapsulated in large multivesicular liposomes (Bupisomes) embedded in hydrogel. This formulation (Bupigel) prolonged bupivacaine release from the formulation in dissolution-like studies in vitro and analgesia in vivo in mouse, rat, and pig models. In this study we assessed Bupigel neurotoxicity on rabbit sciatic nerve using histopathology and electrophysiologic testing. Sciatic nerves of both hind limbs were injected dropwise with different formulations. Nerve conduction studies and needle electromyography two weeks after perineural administration showed signs of neural damage after injection of free lidocaine and bupivacaine, while there was no sign of neural damage after injection with saline, demonstrating the validity of the method. This test also did not show evidence of motor or sensory nerve damage after injection with liposomal bupivacaine at a dose 10-times higher than free bupivacaine. Histologically, signs of neural damage could be observed with lidocaine. Nerves injected with Bupigel showed mild signs of inflammation and small residues of hydrogel in granulomas, indicating a long residence time of the hydrogel at the site of injection, but no histopathological signs of nerve damage. This demonstrated that early signs of neural damage were detected electrophysiologically, showing the usefulness and sensitivity of electrodiagnostic testing in detection of neural damage from new formulations.

## 1. Introduction

Providing effective pain management is a clinical imperative for every patient undergoing surgery. Infiltration of local anesthetics (LAs) into the surgery site is one aspect of the multimodal approach to pre- and postsurgical analgesia. However, the duration of action of LA is limited, lasting only a few hours, and patients may experience breakthrough pain before they are able to take or tolerate oral analgesics, necessitating the use of strong parenteral analgesics (frequently opioids) in the immediate postsurgical period.

Another limitation of the clinical application of LAs is their systemic toxicity, including cardiac and neurological toxicity. The occurrence of life-threatening adverse events related to local anesthetic systemic toxicity has been increasing in recent years [1], highlighting the critical need for long-acting local anesthetics that extend the anesthetic effect while limiting the dose administered to patients, and thus risks associated with their use as well as the need for additional opioids. The cardiotoxic and neurotoxic effects of LAs have been known for some time [2,3] and are dose dependent, but the severity of the phenomenon is different for each LA. The more potent local anesthetics (such as etidocaine and bupivacaine) are for example more cardiotoxic than lidocaine. Liposomal formulations can protect from such negative secondary effects. For instance, encapsulating doxorubicin in PEGylated liposomes significantly reduced the risks of cardiotoxicity associated with the use of free doxorubicin [4,5]. We previously described a formulation of large multivesicular liposomes for the local slow release of bupivacaine [6,7]. Encapsulating bupivacaine into large multivesicular vesicles with a very large trapped aqueous volume, combined with the transmembrane ammonium ion gradient-driven bupivacaine loading, offers several benefits over the free drug. These advantages include higher drug-to-lipid ratio [8,9], a significantly slower release rate [7], producing a much longer duration of anesthesia [9,10] and lower plasmatic C_max_ [6], resulting in lower systemic toxicity. However, the neurological toxicity of this formulation has not been previously addressed. 

The local neurotoxic effects of different LAs have been evaluated in in vitro studies [11,12] and it was shown that all local anesthetics are neurotoxic in a dose-dependent (or concentration-dependent) manner [13] and that clinical levels of the drugs are enough to cause nerve injury [14]. In addition, the duration of exposure to LA also increases the extent of nerve damage [11,13], and the neurotoxicity of LA can be caused by the active drug itself or its additive. Epinephrin is often used together with LA to increase nerve block duration via its vasoconstrictive effect, but the vasoconstriction and resulting decrease in blood flow contribute to prolonging the contact of the nerve with concentrated LA, which may induce damage to the nerve and surrounding tissue [15,16]. In addition, the risk of mechanical damage to the nerve with a needle further increases the risk of neurotoxicity; intrafascicular injection of LA can expose nerves to high concentrations of local anesthetics and increase the associated neurotoxic effects [17].

The objective of this study was to assess neurotoxicity resulting from a long residence time of a local anesthetic in proximity to the sciatic nerve in rabbits. To achieve this goal, we performed a functional neurological test on the sciatic nerve of rabbits following administration of different formulations in this area. Measurements included quantification of nerve conduction velocity (NCV), compound muscle action potentials (CMAPs) amplitude and area, and detection of sural sensory nerve action potentials (SNAPs).

## 2. Materials and Methods

### 2.1. Materials

For the preparation of the liposomal bupivacaine formulation (Bupigel), HSPC (hydrogenated soybean phosphatidylcholine) and cholesterol were purchased from Lipoid GmbH (Ludwigshafen, Germany). The HSPC used in this study is a mixture of two phosphatidylcholines (PCs) containing mostly distearoyl PC (DSPC) mixed with smaller amounts of 1-palmitoyl-2-stearoyl phosphatidylcholine (PSPC). The small batch-to-batch variations in the PSPC/DSPC mole % ratio range from 28/72 to 34/66 and the phase transition temperature (Tm) varied accordingly from 53.04 to 51.07 °C (see Supplementary Table S2 of [18]). The drug product bupivacaine HCl was purchased from MOEHS (Barcelona, Spain) as a powder. For the “free bupivacaine” formulations tested (see Section 2.6. Perineurial Administration Procedure), bupivacaine HCl 5 mg/mL was purchased from Kamada Ltd., Israel and diluted at the desired concentration in ultrapure water. Exparel^®^ (liposomal bupivacaine) was purchased from Pacira Pharmaceuticals (Parsipanny, NJ, USA) and lidocaine HCl (Xylocaine, App Pharmaceuticals, Schaumburg, IL, USA) was purchased as powder and dissolved in ultrapure water at 40 mg/mL (4%). Sterile saline for injection was purchased from Teva (Jerusalem, Israel). 

### 2.2. Preparation of Bupigel

Multilamellar vesicles (MLVs) were prepared as described previously [7]. Briefly, cholesterol and HSPC were dissolved in ethanol at a weight ratio of 4:6. The lipids were then hydrated by adding this ethanolic lipid solution to 250 mM ammonium sulfate. Following incubation for 1 h at 65 °C, the MLVs were submitted to 10 freeze–thaw cycles (2 and 5 min, respectively, in liquid nitrogen and hot water) to increase the trapped aqueous volume of the liposomes. This procedure creates large multivesicular vesicles (LMVVs) [7,9]. A transmembrane ammonium gradient, the driving force for the active remote loading of bupivacaine into LMVVs, was created by replacing the external ammonium sulfate aqueous phase by 0.9% NaCl (saline) with 10 consecutive cycles of centrifugations and suspension of the precipitate with sterile saline. This step also allowed the complete removal of the ethanol that was in the external medium (as was confirmed by osmolality measurements of the external medium that gave results similar to saline osmolality, i.e., 287 ± 2 mOsm/kg). Osmolality was measured with a Model 1332 osmometer (Advanced Instruments, Norwood, MA, USA). Remote active loading of bupivacaine was performed by adding the LMVVs to 5.75% (weight) bupivacaine HCl solution to form Bupisomes. The free (non- encapsulated) drug was then removed from the external medium by centrifugation and replacement of the upper phase by sterile saline. The Bupisomes were then mixed at 1:1 volume ratio with alginate 1% and dropped into a solution of calcium chloride 1.54% to form Bupigel [7]. 

Identical Bupisomes were embedded in other hydrogel-forming agents in addition to alginate 1% to compare their respective bupivacaine release profile (in vitro studies only): Polyvinylpyrrolidone 20% (Kollidon^®^ 25), Poloxamer (Pluronic^®^ F-68), hyaluronic acid 0.5% and alginate 0.3% mixed with CaCl_2_ in stoichiometric ratio. 

### 2.3. Characterization of the Bupisomes and Bupigel

The phospholipid content of Bupisomes was measured by a modified Bartlett procedure as described earlier [19,20]. The bupivacaine content of the formulation (free and encapsulated) was quantified using a Hewlett-Packard series 1100 high-performance liquid chromatography (HPLC) with UV detection. The samples were injected into a 150 × 4.6 mm column (Luna, Phenomenex OOF-4252-EO). A mobile phase of acetonitrile:phosphate buffer 25 mM pH 6.8 (70:30) was used, and absorption was measured at a wavelength of 200 nm. The retention time of the bupivacaine was approximately 5.5 min. The lipid content and encapsulated bupivacaine concentration were used to calculate the drug to lipid ratio. The Bupisomes size was determined using a laser diffraction particle size analyzer (Beckman Coulter LS 13 320). The trapped LMVV aqueous volume of 17.4 mL/mmole HSPC was determined from the intraliposome ammonium concentration [21]. A more detailed physicochemical characterization of Bupigel is described in Table 3 of [7].

### 2.4. “Dissolution” Test for Bupivacaine Release from Bupigel

Bupivacaine release from Bupigel was measured using a standard Apparatus type 2 pharmaceutical dissolution tester Vision G2 Classic 6 (Hanson, Chatsworth, CA, USA) in a dissolution-like approach. The apparatus was equipped with an in-house-designed enhancer cell containing 0.5 g of the sample with a 0.2 µm membrane that allowed only for the low molecular weight molecules to cross without any barrier to the upper compartment containing the desired dissolution buffer (in our case, filtered saline 0.9%). This modified instrument allows close simulation of subcutaneous depot injection. The release tests were performed over 48 h at 37 °C. The concentration of the released drug was quantified in samples (0.5 g) collected from the upper compartment in duplicates at different time points using the same HPLC protocol as described in the section “Characterization of the Bupisomes and Bupigel” above.

### 2.5. Animals

Twenty-three male New Zealand White (NZW) rabbits (strain HsdOkd:NZW, Envigo, Denver, PA, USA) weighing 2.8–3.8 kg at the beginning of the study were allowed to acclimate in the animal facility for at least one week before the study. The rabbits were housed by pairs in 12/12 h light/dark period and allowed free access to water and standard chow (Teklad global rabbit diet) with supplement of fresh hay every 3 days. All in vivo experiments were approved by the Animal Ethical Care Committee of the Hebrew University of Jerusalem, Israel (ethics approval MD-12-13079-3).

### 2.6. Perineurial Administration Procedure

On the day of the nerve block, each rabbit was weighed and anesthetized with an intramuscular (IM) injection of ketamine/xylazine (150/15 mg/kg). They were then maintained under anesthesia by inhalation of isoflurane 2–4% (according to need) until the end of the procedure and kept warm with an electric heating pad. The skin of the rabbit was shaved from the buttock to the knee on both legs. The rabbits were administered antibiotics (cefamezin 30 mg/kg, intramuscular) before surgery (prophylactic) and the 2 following days. They were also injected with a systemic painkiller (carprofen 7.5 mg/kg, subcutaneous, SC) before the beginning of the procedure and for 3 additional days. All IM and SC injections were performed as far as possible from the sciatic nerve in order not to interfere with the experimental measurements.

For perineurial administration, muscles along the planes of fascia between the hamstring muscles were carefully separated in order to expose the sciatic nerve. Each tested formulation (detailed below) was then administered dropwise directly onto the exposed sciatic nerve (in mid-thigh), then the fascia and skin were closed with absorbable suture (Vicryl 3/0). After the procedure rabbits were monitored until they fully awoke and recovered from the surgery. 

The formulations were tested as follows (n being the number of legs injected): saline (200 or 400 µL, *n* = 5) was used as a negative control, while lidocaine 4% (4 mg in 100 µL, *n* = 6) was used as a positive control for nerve damage. Bupigel was tested at 2 doses (5.32 mg and 7.5 mg in 250–400 µL according to the concentration of the formulation, *n* = 4 and *n* = 2, respectively) and compared to free bupivacaine (0.5 to 2 mg, 0.5–2% in a volume of 50–200 µL, *n* = 6, one leg per dose/concentration tested) and to Exparel^®^ (2.66 mg in 200 µL, *n* = 4). In addition, nerve conduction studies (NCSs) were conducted on naïve rabbits (*n* = 6 legs tested) before perineurial administration, in order to compare values of non-injected nerves to saline administration. 

### 2.7. Clinical Follow-up of the Animals

The animals underwent clinical inspection prior to and throughout the study. The rabbits were weighed on the day of perineurial administration and following the surgery, for 3 consecutive days. The incision site was inspected for signs of infection, opening of the sutures or signs of pain. Until the completion of the study, side cage observation was performed daily, but rabbits were weighed only before perineurial administration and NCS in order to decrease stress caused to the animals and especially to minimize risk of hematoma due to leg trauma.

### 2.8. Nerve Conduction Study (NCS)

Two weeks after perineurial drug administration, the rabbits underwent NCSs. For this test, each rabbit was anesthetized with an intramuscular injection of ketamine/xylazine (150/15 mg/kg) in the paraspinous muscle, along the vertebral column (not in the legs in order to avoid the sciatic nerve) and injected with carprofen 5 mg/kg before the beginning of the procedure.

Sciatic-tibial and sciatic-peroneal motor NCSs were tested in both hind limbs, adapted from previously described methods [22,23,24,25,26]. A Dantec Keypoint^®^ Net version 2.11, Natus Medical Incorporated, Skovlunde, Denmark, EMG (electromyography) system was used set at sensitivity 2 mv/div., sweep speed 1 ms/div., HFF 5 KHz, and LFF 10Hz for nerve stimulation and data acquisition. The anesthetized rabbit was placed on a heating pad in a temperature-controlled room in the lateral decubitus position. Rectal temperature was recorded with a thermistor probe and maintained between 38–40 °C (normal values). Thigh skin temperature was monitored using an infrared thermometer (Extech instruments). Fur was shaved on the skin surface and recording 10 mm gold-plated EEG cup electrodes were affixed to the skin in a belly-tendon montage using Ten20 Paste as conductive adhesive and strapped with adhesive tape. A pick-up (G1) cup electrode was placed over the *gastrocnemius* muscle mid-belly for the sciatic-tibial nerve. For sciatic-peroneal nerve, G1 was placed over the *tibialis anterior* muscle at a point along a line 40 percent of the distance measured from *patella* mid-point to lateral ankle. A reference electrode (G2) was placed over the anterior ankle midway between the medial and lateral *malleolus* (peroneal) and *Calcaneus* tendon (tibial). Stimulation was by subdermal disposable sensory needle electrodes (SNEs) (Natus^®^ Alpine bioMed, 28G, 15mm × 0.35 mm) with stimulating cathode (negative pole) and anode 1.5 cm apart. Distal stimulation ≥2.5 cm below the injection site was performed for peroneal nerve at the fibular head level through the upper fibers of the lateral head of *gastrocnemius* muscle, and tibial nerve in the posterior thigh just above the popliteal crease in the midline. Proximal stimulation was at sciatic notch level (≥2.5 cm above injection site). A disposable monopolar needle (Medtronic^®^, 26G 37 mm × 0.4 mm) was placed subcutaneously between stimulator cathode and active pick-up (G1) electrode for grounding. Maximal CMAP amplitudes were obtained both proximally and distally by stepwise increment in stimulation intensity, and without artefacts (currents: 15–80 mA, duration: 0.1–0.2 ms). Supramaximal CMAP amplitude was acquired by further increase in stimulation intensity, without further increase in CMAP size. Measurements obtained were: peak-to-peak CMAP amplitudes (supramaximal), compound muscle action potential area under the negative peak, CMAP duration from onset of the initial negative peak to return to baseline of the final negative peak, and compound muscle action potential latencies measured from stimulus artefact to initial onset of the wave, both proximally and distally. Nerve conduction velocity (NCV) was calculated by dividing the distance by the difference between proximal and distal latencies. Distances were measured by a vinyl inelastic tape measure (Dantec™, Natus^®^ Neurology) along the path of the sciatic-tibial and sciatic-peroneal nerves, between the cathodes of sciatic-notch and distal stimulation sites.

Sural sensory studies were tested from the foot by a method adapted from [23]. The rabbit was placed in the lateral decubitus position with test-side up and fur shaved to skin from distal posterior leg to lateral foot. Stimulation was performed using SNEs as described above placed ~3 cm proximal to the lateral *malleolus* in a groove anterior to *Calcaneus* tendon, cathode-anode 1.5 cm apart. Active pick-up (G1) was from a subdermal SNE inserted in the lateral edge of the foot, 0.5 cm distal to a line perpendicular to lateral edge of foot connecting with lateral *malleolus* above. Reference (G2) SNE was placed 4 cm distal to G1 along the lateral edge of the foot. A ground (monopolar needle electrode) was inserted subcutaneously in the lateral heel. A train of 20–40 SNAPs were averaged, and best of 2 trials taken with filters same as for motor, sweep 2 ms/division and sensitivity 10–20 μV/division. Sural SNAP (sensory nerve action potential) peak latency and amplitude were measured. 

Needle EMG studies were conducted using the above Dantec Keypoint^®^ EMG system with sensitivity 0.1 mv/div., sweep speed 20 ms/div., HFF 5 KHz, and LFF 10 Hz. A disposable concentric needle EMG electrode (0.30 × 25 mm, 30G; Dantec, Alpine Biomed ApS, Skovlunde, Denmark) was inserted into *gastrocnemius* and *tibialis anterior* muscles of both hind limbs to assess for active denervation: fibrillation potentials, positive sharp waves, and other abnormal spontaneous activity. Motor unit action potential analysis was limited due to anesthesia. At the end of the NCS/EMG test, rabbits were sacrificed and sciatic nerves collected and fixed in formalin 4% for histological studies.

### 2.9. Statistical Analysis

All values are expressed as the mean ± standard deviation (SD) unless otherwise stated. Mann–Whitney test was performed using IBM SPSS 25 to compare the saline and Bupigel (5.32 mg) groups, and *p* < 0.05 was the threshold set for statistical significance.

## 3. Results

### 3.1. Characterization of the Formulations and Bupivacaine Release 

The lipid content of the Bupisomes was measured and found to be 6.2 and 16.7 mg/mL for cholesterol and HSPC respectively (total lipid content 22.9 mg/mL). The total bupivacaine content was 2.3% for Bupisomes and 1.8% for Bupigel and the measured free drug concentration in the extravesicular medium was 0.8%. Since the extraliposomal medium (mainly interstitial volume) of Bupisomes was 26.9%, this means that the free drug represents 0.21% of the total formulation volume and the liposome encapsulated/free bupivacaine ratio is 10.95. This low level of the free drug is available immediately and ensures an immediate analgesia. The dissolution tests of both Bupisomes (red triangles) and Bupigel (orange diamonds) at first time point (considered as T = 0) suggest that the levels of free drug is even lower than 0.21%. The calculated drug to lipid mole ratio was 1.86 for the Bupisomes formulation and 1.46 for the Bupigel. The Bupisomes’ average diameter was 15.36 (±1.60) µm. 

**Figure 1:**
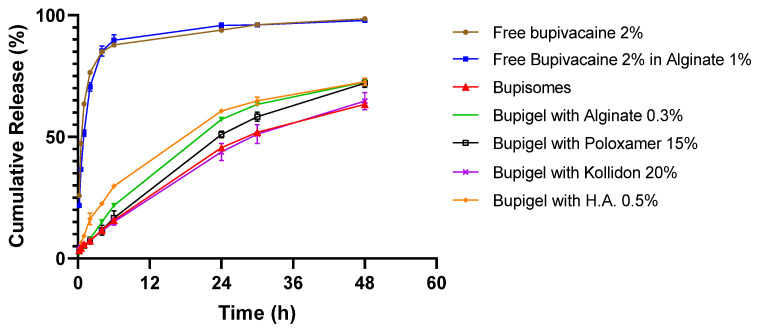
Bupivacaine release profiles from Bupisomes and formulations with different gel-forming agents. The different formulations were incubated at 37 °C under stirring in a standard pharmaceutical dissolution apparatus mimicking subcutaneous depot injection. Bupivacaine was quantified from duplicates of each sample from the upper cell at different time points by HPLC (high-performance liquid chromatography; mean ± SD). H.A., hyaluronic acid.

Results of the release test (Figure 1) show that all the formulations containing Bupisomes exhibit prolonged release compared to free bupivacaine, either in solution or in hydrogel. The Bupisomes in hydrogel formulations released only 63–72% of their drug content over 48 h while the free bupivacaine formulations reached similar release values (69–76%) after 2 h, and above 90% release occurred after 5 h. The release test was stopped after 48 h but the various formulations of hydrogels with Bupisomes still had between 25 and 30% of their bupivacaine content encapsulated and available for continuation of release. The alginate hydrogel had no effect on the free bupivacaine release rate and it did not seem to prolong the release from Bupisomes either, but the hydrogel kept the formulation containing the Bupisomes at the injection site without much movement from the site of injection into adjacent tissue.

### 3.2. Clinical Signs and Body Weight

**Figure 2:**
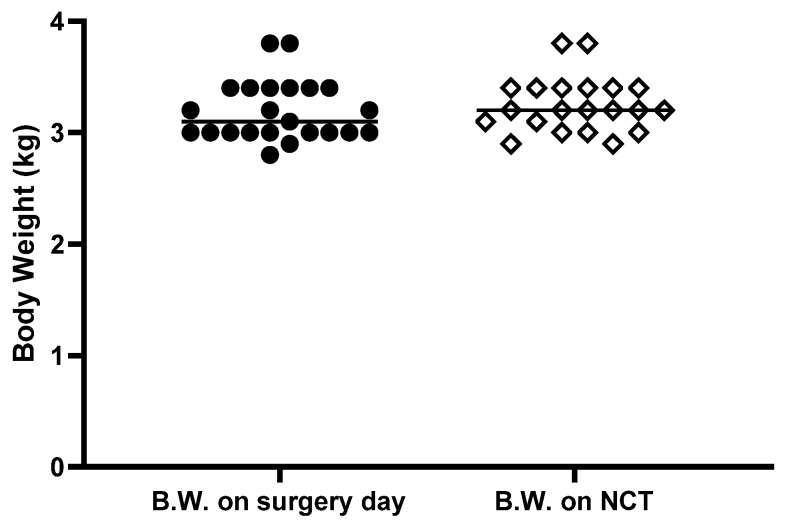
Body weight of the rabbits between the perineurial administration and the nerve conduction study (NCS). Rabbits were weighed before the perineurial administration of the formulations and allowed to recover for 2 weeks before being weighed again and subjected to the NCS.

During the 2 weeks between the perineurial administration and the NCS, rabbits were monitored daily and we observed no behavioral changes during this period. Even just after the surgery they showed no sign of altered gait (such as limping). This is not surprising since rabbits are prey animals, and as such will generally hide signs of pain. For this reason, we also looked at the body weight, a decrease of which could be a sign of serious discomfort, pain, or infection. On perineurial administration day, the mean body weight of the rabbits was 3.19 ± 0.27 kg and on the day of the NCSs it was 3.25 ± 0.25 kg. Since the rabbits were injected with a random combination of two formulations (one on each hind limb), the body weight values and general clinical signs were examined individually to look for any sign that could be correlated with one specific formulation. Figure 2 summarizes the body weight of the rabbits between the perineurial injection and the NCS. During this period only two rabbits lost weight, one rabbit lost 3.3% and the second 5.9% of initial body weight. After consultation, the veterinarian of the animal facility concluded that a loss of body weight of 3% was negligible (as it can result from body fluid variations) while 5.9% was likely due to mild dehydration. Since these two rabbits shared no common formulation, no conclusion could be drawn and we can conclude that no specific formulation administered to the rabbits was correlated with any significant sign of pain or discomfort to the rabbits.

### 3.3. NCS Data Analysis

The results obtained for the saline-injected rabbits (*n* = 5) were used as control for comparison to the formulations for NCV, CMAP amplitude, and CMAP area. The lower limit of normal values (LLN) obtained for this group was used as a basis for the calculations of nerve damage. Any NCV slowing ≤30% below LLN was considered as mild non-specific slowing, within axonal damage range. Slowing >30% below LLN was considered a sign of demyelination. The same measurements were recorded in naïve rabbits (rabbits who did not undergo any type of procedure) in order to get an informative range of the physiological values of nerve conduction in the absence of any procedure.

### 3.4. Nerve Conduction Test: Myelin Parameters

Nerve damage that can occur after administration of the formulations falls into two categories: myelin damage and axonal damage.

Our electrodiagnostic guidelines to identify demyelination were: conduction slowing >30% below LLN, distal latency prolongation >125% of LLN, increased proximal CMAP (sciatic notch) temporal dispersion >25%, and conduction block (>50% drop in proximal CMAP amplitude or area) [26,27,28]. 

**Figure 3:**
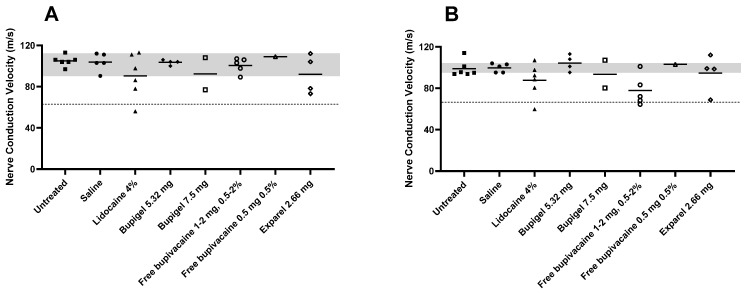
Effect of different formulations on rabbit sciatic nerve conduction velocity (NCV). Individual values for tibial (**A**) and peroneal (**B**) NCV two weeks after perineurial administration of the different formulations. The gray area in the background indicates the range of values for the saline group to facilitate visualization. The dashed line represents the calculated threshold of demyelinative nerve damage (>30% below lower limit of saline value). Saline *n* = 5, lidocaine 4% *n* = 6, Bupigel 5.32 mg *n* = 4, Bupigel 7.5 mg *n* = 2, free bupivacaine 0.5 mg at 0.5% *n* = 1, free bupivacaine (1–2 mg at 0.5–2%) *n* = 5 (1 limb per dose and concentration tested), Exparel^®^ *n* = 4, untreated/naïve *n* = 6, *n* = number of legs.

Figure 3 details the values of tibial (A) and peroneal (B) nerve conduction velocity for each treatment compared to the saline control group range of values and the calculated threshold of demyelinative nerve damage as described above. We can see that the sciatic nerve conduction velocities for the lowest dose of Bupigel tested (5.32 mg, equivalent to twice the dose of Exparel), as well as for the lowest dose of free bupivacaine (0.5 mg at 0.5%) were similar, and within normal range of values (saline control). As expected from the positive control for nerve damage, limbs injected with lidocaine 4 mg 4% had average NCV values, tibial and peroneal, below the LLN. Some limbs had mild slowing (<30% below LLN) which may indicate axonal damage or possible mild demyelination, and others had slowing >30% below LLN, indicating demyelinative damage. A similar result was obtained in limbs injected with free bupivacaine at a dose of 1 mg or more at all tested concentrations. But while the lidocaine-injected limbs exhibited mild non-specific (<30%) slowing (axonal damage range) as well as demyelinative (>30%) slowing in both sciatic peroneal and tibial nerve fibers, the free bupivacaine (1–2 mg, 0.5–2%) group showed demyelinative slowing in the peroneal fibers only.

The limbs injected with Exparel or a high dose (7.5 mg) of Bupigel had similar NCV profiles, showing non-specific mild slowing (<30% below saline LLN), indicating either axonal damage or borderline mild demyelinative damage. In addition, there was moderate slowing (>30% below saline LLN) in the above treated groups in keeping with some mild to moderate demyelination. However, the high standard deviation, due to the small sample size, does not allow us to draw a definitive statistically significant conclusion about this reduction.

Table 1 summarizes additional electrophysiological findings for the demyelinative nerve damage parameters, namely NCV average, number of limbs with NCV 30% below LLN, presence or absence of conduction block, and presence or absence of proximal CMAP increased temporal dispersion (>25%).

### 3.5. Nerve Conduction Tests: Axonal Parameters

Axonal damage can be measured by: (1) A decrease in distal CMAP amplitude and area compared to lower limit of normal of saline group, with or without mild NCV slowing (≤30% below LLN), or absent CMAP; (2) Absence of sural SNAP amplitude; (3) Active denervation on needle EMG (fibrillation potentials, positive sharp waves). In this study, the motor unit action potential recruitment or volition was not assessed because the test was performed under general anesthesia. Needle EMG is more sensitive to mild degrees of denervation when the CMAP amplitude is within the LLN [28].

**Figure 4:**
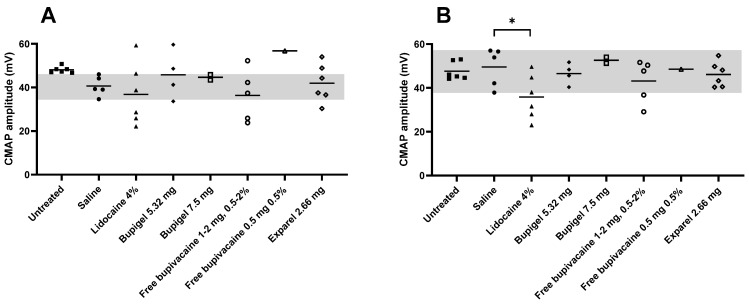
Effect of the different formulations on distal CMAP (compound muscle action potentials) amplitudes. Tibial (**A**) and peroneal (**B**) CMAP amplitudes were measured two weeks after perineurial administration of the different formulations. Gray area in the background indicates the range of values for the saline group. Any value below the saline group’s LLN (gray area) was considered as abnormal (axonal damage). Saline *n* = 5, lidocaine 4% *n* = 6, Bupigel 5.32 mg *n* = 4, Bupigel 7.5 mg *n* = 2, free bupivacaine 0.5 mg at 0.5% *n* = 1, free bupivacaine (1–2 mg at 0.5–2%) *n* = 5 (1 limb per dose and concentration tested), Exparel^®^ *n* = 4, untreated/naïve *n* = 6. * *p* < 0.05, *n* = number of legs.

**Figure 5:**
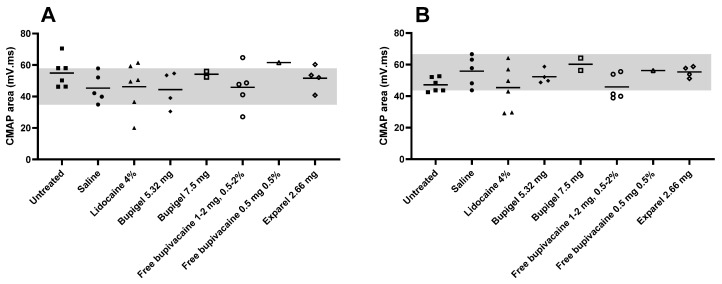
Effect of the different formulations on distal CMAP areas. Individual tibial (**A**) and peroneal (**B**) CMAP area values were measured two weeks after the perineurial administration of the different formulations. The gray area in the background indicates the range of values obtained in the saline group. Saline *n* = 5, lidocaine 4% *n* = 6, Bupigel 5.32 mg *n* = 4, Bupigel 7.5 mg *n* = 2, free bupivacaine 0.5 mg at 0.5% *n* = 1, free bupivacaine (1–2 mg at 0.5–2%) *n* = 5 (1 limb per dose and concentration tested), Exparel^®^ *n* = 4, untreated/naïve *n* = 6.

Figure 4 details the individual formulations’ distal CMAP tibial (A) and peroneal (B) amplitudes compared to saline (control) for threshold of axonal damage, while Figure 5 details the individual values for distal CMAP areas. Tibial CMAP amplitude (Figure 4) was characterized by a wide range of values, while peroneal fibers had lower standard deviations. In both sciatic tibial and peroneal motor fibers, three lidocaine-injected limbs displayed a substantial drop in amplitude, but due to the high standard deviation (and small number of tested legs), only the decrease in the peroneal motor fibers in the lidocaine group was significantly lower than the saline group. Of note, in the saline group, mild sciatic motor nerve axonal impairment was indicated by a lower average tibial CMAP amplitude compared to naïve rabbits, without any increased temporal dispersion. However, this was not confirmed by a drop in CMAP area nor active denervation on needle EMG, suggesting this finding was either possibly due to mild axonal damage or the low sample size. In proximal CMAP areas (Figure 5), no statistical significance could confirm differences between the average of the different groups because of the high standard deviation due to the small sample size.

Table 2 summarizes the parameters of axonal damage (average distal CMAP amplitude and area as well as the number of limbs in each group, EMG signs of active denervation, or absent sural SNAPs). In this table we can see that only rabbits in the lidocaine-injected group presented signs of active denervation (one rabbit had mild tibialis anterior denervation while another had signs of moderate denervation in the gastrocnemius muscle). In addition, sural SNAPs were absent (a sign of sensory axonal damage) in the lidocaine group (3 out of 5 legs), Exparel group (3 out of 4 legs), and in all the legs of rabbits injected with doses of free bupivacaine ≥ 1 mg and high dose (7.5 mg) of Bupigel.

Table 3 summarizes all signs of nerve damage measured after the administration of the different formulations. 

### 3.6. Histology

**Figure 6:**
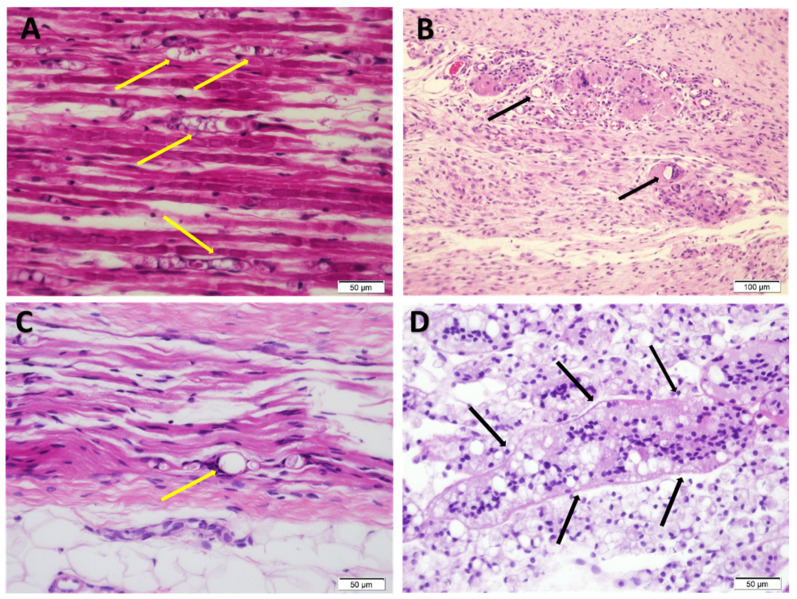
Sciatic nerve at the injected site 2 weeks after perineurial injection of lidocaine 4 mg 4% (**A**), Bupigel 5.32 mg (**B**,**C**) or Bupigel 7.5 mg (**D**). The yellow arrows indicate ovoids, consistent with Wallerian-like degeneration and the black arrows point to foreign body type granulomas (H&E staining, ×40 (A, C and D) or ×20 [B]).

The samples of the sciatic nerve around the injection site were collected immediately after NCSs (2 weeks after perineurial injection). In the legs injected with saline and with free bupivacaine (1 mg at 0.5%), the presence of few mononuclear cells around the blood vessels indicated mild inflammation. In contrast, the legs injected with lidocaine (4 mg at 4% solution) showed many ovoids as a sign of Wallerian-like degeneration (Figure 6A) consistent with axonal injury. The limbs injected with Exparel^®^ exhibited much milder Wallerian-like degeneration, with only a few ovoids. In the samples from legs injected with the lowest dose of Bupigel (5.32 mg), foreign body type granulomas containing particles of hydrogel could be observed in the epineurium (Figure 6B). Very few ovoid-like structures were identified, but since these structures were localized only adjacent to the perineurium, the possibility of foreign body type granulomas cannot be entirely excluded (Figure 6C). In the samples injected with the high dose of Bupigel (7.5 mg), the foreign body type granulomas were more pronounced in the epineurium (Figure 6D). This shows that the clearance of the hydrogel was slow, indicating long residence time of the Bupigel formulations at the site of injection. Overall, the observations showed signs of inflammatory response to the injection of Bupigel, but no obvious indication of nerve injury per se.

## 4. Discussion

Our study shows that neurotoxic effects of local anesthetics can be measured by NCS, a functional test performed two weeks after local injection. Electrodiagnostic testing (nerve conduction and needle EMG) allows for a non-invasive and detailed characterization of a neuropathy. It is highly sensitive for defining the pattern and degree of nerve involvement and can provide insight into the underlying pathophysiology, defining a neuropathy as either axonal or demyelinating, and gives an idea of the severity of the damage. However, because it is so sensitive, the results are affected by anything between the electrodes and the nerve, such as peripheral edema resulting from trauma [29]. Rabbits are very sensitive to stress and therefore they tend to run and kick if they feel aggressed, for example in the presence of an aggressive cage mate, and any blow or bite to the region tested (from the thigh to the ankle) may cause edema that can disturb the NCS readings. For this reason, great care must be taken in the handling and housing of the rabbits. Contacts between the animals and caretaker should be maintained to a minimum, and male rabbits should be housed individually if they show signs of aggressiveness to avoid local trauma [30]. In addition, drugs other than the tested formulations that require intramuscular injection (such as painkillers, antibiotics, or drugs used to induce anesthesia) should not be administered close to the tested area because any pressure caused by the volume of injection in the nearby muscles can modify nerve conduction. The effect on the NCS is obviously more important if the formulation is targeting the nerve. This was observed when the induction of anesthesia in rabbits was performed by an intramuscular injection of ketamine and xylazine in the femoral biceps without any other procedure. The injection of the local anesthetic in muscles close to the tested area caused very severe partial conduction block in both sciatic-tibial and sciatic-peroneal motor fibers, with motor NCV slowing and moderately severe secondary axonal damage (ongoing/active denervation). In addition, sural sensory studies were absent (data not shown). For this reason, the cocktail of anesthesia inducers was injected as far as possible from the sciatic nerve, in the dorsal area, for the rest of the study. In addition, a study [31] showed that the use of ketamine and xylazine as anesthetic agents caused a decrease in motor NCV values in mice (while sensory NCV values remained unchanged) compared to animals anesthetized with isoflurane. In our study, the rabbits received a dose of ketamine and xylazine to induce the anesthesia while the procedure itself (perineurial administration and NCS) was performed under isoflurane anesthesia. Nevertheless, the NCV decrease observed in mice was reported to be consistent within experimental groups and across multiple experiments [31], therefore it should not be of concern in this study. Another effect observed by the same group following ketamine and xylazine anesthesia in mice is a decrease in core and surface temperature (measured in hind limbs) compared to mice anesthetized with isoflurane. It is widely known that temperature affects diverse parameters of NCS [32,33,34,35], and for this reason the rabbits’ temperatures were maintained constant during the procedure with a heating pad. In addition, NCS requires the animals to be under general anesthesia, thus restricting needle EMG motor unit action potential analysis and volition/recruitment. However, performing the NCS on the sciatic nerve is an advantage because of the large perineurial space, allowing the administration of relatively large volumes of formulations without causing pressure on the nerve. For this reason, the lower tibial CMAP amplitude observed in saline-injected legs compared to naïve (untreated) is unlikely due to mechanical compression of the nerve by the volume of saline injected but rather could be caused by irritation caused by saline touching the nerve and triggering vasomotor responses. The effect of saline has been studied in epineurial injections [36,37] and has been shown to cause nerve damage. Despite the use of saline-injected legs as control, there has been no NCS comparison in naïve animals compared to perineurial injection of saline to the best of our knowledge. 

The interval between the perineurial injection and the NCS (2 weeks) was chosen considering several parameters. The first parameter is the rate of drug release determined by dissolution kinetics (Figure 1) and from plasma PK studies in humans [6] where the long half-life of Bupisome injected SC was found to be 294 ± 860 min compared to 131 ± 58 min for free bupivacaine 0.5%. The second parameter is having the NCS 14 days after the perineurial injection, giving sufficient time to detect any electrophysiological evidence of nerve damage. The third point to take into consideration is that the NCS should be performed after the resorption of post-operative inflammation (one week after perineurial administration, the inflammation in the legs was still impairing NCS measurements, data not shown). The fourth and final parameter is the presence of local anesthetic near the sciatic nerve that will, by nature, slow nerve impulses and therefore affect the NCS. Thus, sufficient time should be given to allow for complete wash-out of the tested formulations. In adults, the terminal half-life of bupivacaine is 2.7 h [38]. Therefore, 2 weeks (the interval between the administration of local anesthetic and the nerve conduction study), is sufficient for complete wash-out of all tested formulations, and the variations in the different parameters of NCS measured are due to nerve damage and not to the direct effect of the local anesthetic on the nerves.

A study [39] assessing the neurotoxicity of a liquid formulation of liposomal bupivacaine after perineural administration in pigs showed that even using electron microscopy, there was no detectable sign of neurotoxicity caused by the formulation. Signs of inflammatory response were detected, as expected after a surgical procedure. Similarly, in our study, microscopic examination did not reveal signs of nerve injury besides mild signs of inflammation that are most probably residues of post-operative inflammation. In addition, this effect was only observed in sciatic tibial CMAP amplitude but not in the peroneal fibers, nor in CMAP area or nerve velocity. Similarly, no effect of saline could be detected by active denervation on needle EMG, suggesting any possible axonal damage was only mild and limited to tibial fibers.

In our study the positive control for nerve damage (lidocaine 4 mg at 4%) proved to cause nerve damage in four rabbits out of five. The nerve damage in the rabbits affected was mostly mild motor demyelination and three rabbits out of four had axonal damage in the sensory fibers (absent sural sensory SNAP). One of the limbs injected with lidocaine had a mild partial conduction block, indicating the presence of mild focal motor myelin damage.

In order to find the smallest dose (and concentration) of free bupivacaine that would not cause neurotoxicity we tried several combinations: 2 mg at 2% or 1%, 1 mg at 2%, 1% and 0.5%, and finally 0.5 mg at 0.5%. Among all these combinations, only the smallest dose (0.5 mg at 0.5%) did not cause damage to the nerve. The limbs injected with doses of free bupivacaine between 1–2 mg (concentration ranging from 0.5% to 2%) showed varying degrees of nerve damage, from mild non-specific NCV slowing in axonal range (less than 30% below LLN, 64.4 to 72 m/s), to mild demyelinative damage shown by 30–40% slowing below LLN and mild partial conduction block. In addition, there was sensory fiber damage in all cases (5/5), but no motor axonal damage with this preparation. 

The limbs injected with a dose of free bupivacaine as small as 1 mg showed signs of neurotoxicity with mild motor myelin damage and absent sensory potentials. However, we demonstrated in this study than none of the limbs injected with a dose more than 5-times higher of liposomal bupivacaine showed any signs of nerve damage. The limbs injected with 5.32 mg of bupivacaine encapsulated in Bupigel had NCV values that were within the normal range and distal CMAP amplitudes very similar to the limbs injected with saline. There were no signs of motor axonal or sensory fiber damage in the Bupigel-injected limbs at this dose. Increasing the liposomal bupivacaine dose to 7.5 mg resulted in mild motor myelin damage and absent sural SNAPs. This dose is apparently above the toxic threshold for our liposomal formulation. Interestingly, Exparel^®^ administration caused signs of motor myelin and sensory fibers damage at half of the Bupigel dose that was found innocuous. The high standard deviation related to the low number of animals in each group and high variations between individuals did not allow us to draw definitive conclusions regarding the effect of the formulations compared to saline (except in one parameter). Nevertheless, there was an obvious trend showing a toxic effect in several nerves, including clear signs of demyelinative damage in lidocaine-injected legs, showing the relevance of such a test for the detection of neurotoxicity. 

One of the major limitations of LAs is their short duration of action. The duration of anesthesia is in part influenced by the residence time of the LA in close proximity to the neural fibers, and for this reason vasopressors are added to many LA formulations. Their effect, constricting neighboring vasculature, delays the absorption of the LA to the systemic circulation and therefore extends the effect of the anesthesia [40]. The use of our formulation, consisting of large multivesicular liposomes with high trapped aqueous volume which are embedded in hydrogel, overcomes the need for vasopressors in order to keep the LA at the action site. The viscosity of various hydrogel-forming polymers including hyaluronic acid or alginate hydrogels, ensures good injectability and is high enough to mechanically keep the formulation at the injection site where the bupivacaine is slowly released [7]. We previously showed that the slow release offered by liposomal formulation protects from the cardiotoxic effect of free bupivacaine [6]. In this study we demonstrated in addition that Bupigel formulation is innocuous at the tested dose with regard to local toxicity to the sciatic nerve, as shown by the nerve conduction study performed that allowed us to administer a dose more than 5-times higher than the non-liposomal bupivacaine without any sign of local nerve damage. In addition, performing a functional test for the detection of neurotoxicity allowed us to detect neural damage that could not always be observed by traditional histological observations.

## 5. Conclusions

Bupigel (liposomal bupivacaine in hydrogel), injected at a dose of 5.32 mg per site, did not cause significant nerve toxicity. This dose is equivalent to 10-times the highest dose of free bupivacaine that was not neurotoxic. We also showed that the NCS is a sensitive functional test that allows the detection of early signs of neurotoxicity without the need to sacrifice the animal for histology.

## 6. Patents

Yechezkel Barenholz is one of the inventors of Barenholz Y., Cohen R.: “Composition of matter comprising liposomes embedded in a polymeric matrix and methods of using same” US patent 9,713,591 B2 25 July 2017. This patent was licensed in 2014 by Yissum, the TTO of the Hebrew University of Jerusalem to LipocureRX which sublicensed it to Virpax Inc in March 2018, to further develop it.

## Figures and Tables

**Table 1:** Summary of electrophysiological findings for sciatic motor nerve myelin parameters.

Treatment	Nerve	NCV (m/s)	No. >30% Slowing below LLN (Myelin Damage)	No. with Conduction Block	No. with Increased prox. CMAP Dispersion (>25%)
Naïve (no injection) *n* = 6	Tibial	105.00 (±5.14)	0/6	0/6	0/6
	Peroneal	98.82 (±7.91)	0/6	0/6	0/6
Saline *n* = 5	Tibial	103.88 (±8.66)	0/5	0/5	0/5
	Peroneal	99.68 (±4.23)	0/5	0/5	0/5
Lidocaine 4% *n* = 6	Tibial	90.35 (±21.64)	2/6	1/6	0/6
	Peroneal	87.68 (±16.29)	1/6	0/6	1/6
Bupigel (5.32 mg) *n* = 4	Tibial	103.5 (±2.52)	0/4	0/4	0/4
	Peroneal	104.3 (±7.81)	0/4	0/4	0/4
Bupigel (7.5 mg) *n* = 2	Tibial	92.45 (±21.99)	1/2	0/2	0/2
	Peroneal	93.60 (±18.95)	0/2	0/2	0/2
Free bupivacaine (0.5 mg 0.5%) *n* = 1	Tibial	109.0	0/1	0/1	0/1
	Peroneal	103.0	0/1	0/1	0/1
Free bupivacaine (1–2 mg, 0.5–2%) *n* = 5	Tibial	100.60 (±7.28)	0/5	0/5	0/5
	Peroneal	77.8 (±14.78)	4/5	1/5	0/5
Exparel (2.66 mg) *n* = 4	Tibial	91.90 (±18.99)	2/4	0/4	0/4
	Peroneal	94.60 (±18.29)	1/4	0/4	0/4

**Table 2:** Summary of electrophysiological findings for sciatic nerve axonal parameters.

Treatment	Nerve	Distal CMAP Amplitude (mV)	Distal CMAP Area (mV.ms)	Active Denervation	Absent Sural SNAP
Naïve (no injection) *n* = 6	Tibial	48.07 (±1.48)	54.87 (±9.32)	0/6	0/6
	Peroneal	47.60 (±4.08)	47.15 (±4.51)		
Saline *n* = 5	Tibial	40.60 (±4.52)	45.36 (±9.35)	0/4 *	0/5
	Peroneal	49.50 (±8.88)	55.84 (±9.74)		
Lidocaine 4% *n* = 6	Tibial	36.83 (±14.17)	46.2 (±15.57)	2/5 *	3/5 *
	Peroneal	35.83 (±10.18)	45.37 (±14.29)		
Bupigel (5.32 mg) *n* = 4	Tibial	45.75 (±11.08)	44.38 (±11.65)	0/4	0/4
	Peroneal	46.53 (±4.78)	52.30 (±4.50)		
Bupigel (7.5 mg) *n* = 2	Tibial	44.65 (±1.77)	54.15 (±2.62)	0/2	2/2
	Peroneal	52.60 (±1.98)	60.15 (±5.44)		
Free bupivacaine (0.5 mg at 0.5%) *n* = 1	Tibial	56.80	61.5	0/1	0/1
	Peroneal	48.50	56.2		
Free bupivacaine (1–2 mg at 0.5–2%) *n* = 5	Tibial	36.34 (±11.81)	45.84 (±13.61)	0/5	5/5
	Peroneal	43.12 (±9.78)	45.86 (±8.10)		
Exparel (2.66 mg) *n* = 4	Tibial	44.25 (±8.58)	51.65 (±8.08)	0/4	3/4
	Peroneal	47.10 (±6.44)	55.30 (±3.50)		

CMAP = compound muscle action potential (average ± SD), SNAP = sensory nerve action potential (given as number of absent studies), active denervation = *tibialis anterior* and /*gastrocnemius* muscle needle EMG showing positive sharp waves/fibrillation potentials. * One rabbit was not tested for technical reasons.

**Table 3:** Summary of signs of nerve damage according to treatment.

Treatment	No. of Nerves with Damage		
	Motor Axonal	Motor Myelin	Sensory (Absent Sural SNAP)
Naïve (no injection) *n* = 6	0/6	0/6	0/6
Saline *n* = 5	0/4 *	0/5	0/5
Lidocaine 4% *n* = 6	2/5 *	3/6	3/5 *
Bupigel (5.32 mg) *n* = 4	0/4	0/4	0/4
Bupigel (7.5 mg) *n* = 2	0/2	1/2	2/2
Free bupivacaine (0.5 mg at 0.5%) *n* = 1	0/1	0/1	0/1
Free bupivacaine (1–2 mg at 0.5–2%) *n* = 5	0/5	4/5	5/5
Exparel (2.66 mg) *n* = 4	0/4	3/4	3/4

* One rabbit could not be tested for technical reasons.

## Data Availability

The data presented in this study are available on request from the corresponding author.

## References

[1] El-Boghdadly K., Pawa A., Chin K.J. (2018). Local Anesthetic Systemic Toxicity: Current Perspectives. Local Reg. Anesth..

[2] Reiz S., Nath S. (1986). Cardiotoxicity of Local Anaesthetic Agents. Br. J. Anaesth..

[3] Kyttä J., Heinonen E., Rosenberg P.H., Wahlström T., Gripenberg J., Huopaniemi T. (1986). Effects of Repeated Bupivacaine Administration on Sciatic Nerve and Surrounding Muscle Tissue in Rats. Acta Anaesthesiol. Scand..

[4] Safra T., Muggia F., Jeffers S., Tsao-Wei D.D., Groshen S., Lyass O., Henderson R., Berry G., Gabizon A. (2000). Pegylated Liposomal Doxorubicin (Doxil): Reduced Clinical Cardiotoxicity in Patients Reaching or Exceeding Cumulative Doses of 500 Mg/M2. Ann. Oncol..

[5] O’Brien M.E.R., Wigler N., Inbar M., Rosso R., Grischke E., Santoro A., Catane R., Kieback D.G., Tomczak P., Ackland S.P. (2004). Reduced Cardiotoxicity and Comparable Efficacy in a Phase III Trial of Pegylated Liposomal Doxorubicin HCl (CAELYX^TM^/Doxil^®^) versus Conventional Doxorubicin for First-Line Treatment of Metastatic Breast Cancer. Ann. Oncol..

[6] Davidson E.M., Barenholz Y., Cohen R., Haroutiunian S., Kagan L., Ginosar Y. (2010). High-Dose Bupivacaine Remotely Loaded into Multivesicular Liposomes Demonstrates Slow Drug Release without Systemic Toxic Plasma Concentrations after Subcutaneous Administration in Humans. Anesth. Analg..

[7] Cohen R., Kanaan H., Grant G.J., Barenholz Y. (2012). Prolonged Analgesia from Bupisome and Bupigel Formulations: From Design and Fabrication to Improved Stability. J. Control. Release.

[8] Mantripragada S. (2002). A Lipid Based Depot (DepoFoam^®^ Technology) for Sustained Release Drug Delivery. Prog. Lipid Res..

[9] Grant G.J., Barenholz Y., Bolotin E.M., Bansinath M., Turndorf H., Piskoun B., Davidson E.M. (2004). A Novel Liposomal Bupivacaine Formulation to Produce Ultralong-Acting Analgesia. Anesthesiology.

[10] Grant G.J., Piskoun B., Bansinath M. (2003). Analgesic Duration and Kinetics of Liposomal Bupivacaine after Subcutaneous Injection in Mice. Clin. Exp. Pharmacol. Physiol..

[11] Yang S., Abrahams M.S., Hurn P.D., Grafe M.R., Kirsch J.R. (2011). Local Anesthetic Schwann Cell Toxicity Is Time and Concentration Dependent. Reg. Anesth. Pain Med..

[12] Cai X.Y., Xiong L.M., Yang S.H., Shao Z.W., Xie M., Gao F., Ding F. (2014). Comparison of Toxicity Effects of Ropivacaine, Bupivacaine, and Lidocaine on Rabbit Intervertebral Disc Cells in Vitro. Spine J..

[13] Werdehausen R., Fazeli S., Braun S., Hermanns H., Essmann F., Hollmann M.W., Bauer I., Stevens M.F. (2009). Apoptosis Induction by Different Local Anaesthetics in a Neuroblastoma Cell Line. Br. J. Anaesth..

[14] Selander D., Brattsand R., Lundborg G., Nordborg C., Olson Y. (1979). Local Anesthetics: Importance of Mode of Application, Concentration and Adrenaline for the Appearance of Nerve Lesions: An Experimental Study of Axonal Degeneration and Barrier Damage after Intrafascicular Injection or Topical Application of Bupivacaine. Acta Anaesthesiol. Scand..

[15] Hashimoto K., Hampl K.F., Nakamura Y., Bollen A.W., Feiner J., Drasner K. (2001). Epinephrine Increases the Neurotoxic Potential of Intrathecally Administered Lidocaine in the Rat. Anesthesiology.

[16] Hogan Q.H. (2008). Pathophysiology of Peripheral Nerve Injury during Regional Anesthesia. Reg. Anesth. Pain Med..

[17] Whitlock E.L., Brenner M.J., Fox I.K., Moradzadeh A., Hunter D.A., Mackinnon S.E. (2010). Ropivacaine-Induced Peripheral Nerve Injection Injury in the Rodent Model. Anesth. Analg..

[18] Wei X., Cohen R., Barenholz Y. (2016). Insights into Composition/Structure/Function Relationships of Doxil^®^ Gained from “High-Sensitivity” Differential Scanning Calorimetry. Eur. J. Pharm. Biopharm..

[19] Barenholz Y., Amselem S., Gregoriadis G. (1993). Quality control assays in the development and clinical use of liposome-based formulations. Liposome Technology.

[20] Shmeeda H., Even-Chen S., Honen R., Cohen R., Weintraub C., Barenholz Y. (2003). Enzymatic Assays for Quality Control and Pharmacokinetics of Liposome Formulations: Comparison with Nonenzymatic Conventional Methodologies. Methods in Enzymology.

[21] Cohen R., Steiner A., Kanaan H., Barenholz Y. (2013). Chemical and Physical Characterization of Remotely Loaded Bupivacaine Liposomes: Comparison between Large Multivesicular Vesicles and Small Unilamellar Vesicles. J. Mater. Chem. B.

[22] Chuang T.-Y.Y., Chan R.-C.C., Chin L.-S.S., Hsu T.-C.C. (1995). Neuromuscular Injury during Limb Lengthening: A Longitudinal Follow-up by Rabbit Tibial Model. Arch. Phys. Med. Rehabil..

[23] Chiou-Tan F.Y., Chiou G.C.Y. (2000). Contribution of Circulating Acetylcholine to Sensory Nerve Conduction Augmentation. Life Sci..

[24] Sung D.H., Han T.R., Park W.H., Je Bang H., Kim J.M., Chung S.H., Woo E.J. (2001). Phenol Block of Peripheral Nerve Conduction: Titrating for Optimum Effect. Arch. Phys. Med. Rehabil..

[25] Henry F.P., Goyal N.A., David W.S., Wes D., Bujold K.E., Randolph M.A., Winograd J.M., Kochevar I.E., Redmond R.W. (2009). Improving Electrophysiologic and Histologic Outcomes by Photochemically Sealing Amnion to the Peripheral Nerve Repair Site. Surgery.

[26] Rabie M., Yanay N., Fellig Y., Konikov-Rozenman J., Nevo Y. (2019). Improvement of Motor Conduction Velocity in Hereditary Neuropathy of LAMA2-CMD Dy2J/Dy2J Mouse Model by Glatiramer Acetate. Clin. Neurophysiol..

[27] Nevo Y., Topaloǧlu H. (2002). 88th ENMC International Workshop: Childhood Chronic Inflammatory Demyelinating Polyneuropathy (Including Revised Diagnostic Criteria), Naarden, The Netherlands, 8–10 December 2000. Neuromuscul. Disord..

[28] Bromberg M.B. (2013). An Electrodiagnostic Approach to the Evaluation of Peripheral Neuropathies. Phys. Med. Rehabil. Clin. N. Am..

[29] Whittaker R.G. (2012). SNAPs, CMAPs and F-Waves: Nerve Conduction Studies for the Uninitiated. Pract. Neurol..

[30] Morton D.B., Jennings M., Batchelor G.R., Bell D., Birke L., Davies K., Eveleigh J.R., Gunn D., Heath M., Howard B. (1993). Refinements in Rabbit Husbandry: Second Report of the BVAAWF/FRAME/RSPCA/UFAW Joint Working Group on Refinement. Lab. Anim..

[31] Oh S.S., Hayes J.M., Sims-Robinson C., Sullivan K.A., Feldman E.L. (2010). The Effects of Anesthesia on Measures of Nerve Conduction Velocity in Male C57Bl6/J Mice. Neurosci. Lett..

[32] Hopf H.C., Maurer K. (1990). Temperature Dependence of the Electrical and Mechanical Responses of the Adductor Pollicis Muscle in Humans. Muscle Nerve.

[33] Kiernan M.C. (2001). Effects of Temperature on the Excitability Properties of Human Motor Axons. Brain.

[34] Rutkove S.B. (2001). Effects of Temperature on Neuromuscular Electrophysiology. Muscle Nerve.

[35] Drenthen J., Blok J.H., Van Heel E.B.M.D., Visser G.H. (2008). Limb Temperature and Nerve Conduction Velocity during Warming with Hot Water Blankets. J. Clin. Neurophysiol..

[36] Rayan G.M., Gannaway J.K., Pitha J., Dale G.L. (1985). Peripheral Nerve Changes Following Epineurial Injection of Saline and Blood in Rat Sciatic Nerve. Clin. Orthop. Relat. Res..

[37] Hackel D., Krug S.M., Sauer R.S., Mousa S.A., Böcker A., Pflücke D., Wrede E.J., Kistner K., Hoffmann T., Niedermirtl B. (2012). Transient Opening of the Perineurial Barrier for Analgesic Drug Delivery. Proc. Natl. Acad. Sci. USA.

[38] Bupivacaine Leaflet (FDA). https://www.accessdata.fda.gov/drugsatfda_docs/label/2012/018692s015lbl.pdf.

[39] Damjanovska M., Cvetko E., Hadzic A., Seliskar A., Plavec T., Mis K., Hasanbegovic I.V., Pintaric T.S. (2015). Neurotoxicity of Perineural vs Intraneural-Extrafascicular Injection of Liposomal Bupivacaine in the Porcine Model of Sciatic Nerve Block. Anaesthesia.

[40] Becker D.E., Reed K.L. (2012). Local Anesthetics: Review of Pharmacological Considerations. Anesth. Prog..

